# Cytological characterization and molecular mapping of a novel recessive genic male sterility in sesame (*Sesamum indicum* L.)

**DOI:** 10.1371/journal.pone.0204034

**Published:** 2018-09-20

**Authors:** Hongyan Liu, Fang Zhou, Ting Zhou, Yuanxiao Yang, Yingzhong Zhao

**Affiliations:** Key Laboratory of Oil Crops Biology and Genetic Improvement, Ministry of Agriculture, Oil Crops Research Institute, CAAS, Wuhan, China; The Institute of Genetics and Developmental Biology (IGDB) of the Chinese Academy of Sciences (CAS), CHINA

## Abstract

Recessive genic male sterility (RGMS) has great potential for F_1_ hybrid seeds production in sesame (*Sesamum indicum* L.). However, it is not yet widely used in practice due to poor understanding of the underlying mechanism in RGMS. Previously, we have developed a novel sesame RGMS line (D248A) controlled by a single recessive gene. To elucidate its cytological mechanism, histological observations were carried out in sterile and fertile buds. The results indicated that abnormality in D248A began at microspore mother cell stage and persisted until microspore stage. The microsporocytes had less cytoplasm and no obvious nucleus. Normal meiosis failed in microspore mother cells. Cytoplasm condensation and vacuolation frequently occurred in tetrads, leading to the production of crumpled and abortive microspores. To develop molecular markers for breeding of hybrid lines, InDel and SSR markers were analyzed in a fertility segregating NIL population constructed by sib-mating D248A with D248B. Five markers were identified for the male sterile gene (*Ms*), with a respective genetic distance of 1.47 and 5.17 cM for the two closest markers (SB2993 and LG1-170) on both sides. The *Ms* gene was further anchored into a 108-kb interval in the downstream of chromosome 1, within which 15 genes were predicted and four were likely to be responsible for male sterility. These findings provide a deeper understanding of the mechanism underlying RGMS in sesame.

## Introduction

Sesame (*Sesamum indicum* L.) is a member of the Pedaliaceae family and has long been cultivated for edible oil and cuisine ingredients in tropical and subtropical areas [1,2]. It is such a crop that produces the highest (50–60%) and richest oil in seeds [3]. The oil product has an excellent nutrition value due to the presence of natural anti-oxidative constituents such as sesamol, sesamolinol, sesaminol and γ-tocopherol [4–6]. In addition, sesame is also an easily accessible and affordable source of protein in developing countries [7]. Globally, 6.11 million tons of sesame seeds have been produced on 10.58 million ha land in 2016 (UN Food and Agriculture Organization Statistical Databases, 2016). The top four producers, Tanzania, Myanmar, India, and China, accounted for 52% of the world’s production. However, the average seed yield was only 0.58 metric tons per ha in 2016, which was much lower than many other crops. This low productivity was possibly because most of the currently cultivated homozygous varieties were bred through hybridization and selection [8]. Moreover, with the increasing of living standards and knowledge on nutrition value of sesame oil, the market demand for sesame products grows rapidly and steadily. For instance, sesame seeds imported by China alone increased from 0.19 million tons in 2008 to 0.45 mt in 2013 [FAOSTAT, 2013]. Therefore, substantial improvement for total seed yield and yield per unit should be made to meet the huge demand.

Crop yield improvement can be achieved by several approaches, among which heterosis utilization via genic male sterility (GMS) or cytoplasmic male sterility (CMS) is the most effective and efficient ones. CMS is widely used in the breeding and production of F_1_ hybrids in many crops such as rice and rapeseed [9,10]. High level of heterosis for seed yield and its component traits was also observed in sesame [11–15]; however, exploiting such heterosis is still at its infant stage due to the lack of a suitable pollen controlling system in this highly self-pollinated crop. In many other crops, GMS systems are regarded as a promising alternative to CMS because GMS systems show stable and complete male sterility, no negative cytoplasmic effects, and easy transference of the male sterile genes to diverse genetic backgrounds [10]. In addition, recessive GMS (RGMS) has another advantage over CMS because by which a hybrid with strong heterosis is much easier to breed, since most varieties are restorers for this system [10]. In the pass decays, several sesame RGMS lines have been developed, which were assumed to be controlled by one or two nuclear genes that play a key role in pollen formation and development [16,17]. These included ms86-1 [18], 95ms-5 [19] and 0176A [20], as well as their derivative lines. ms86-1 was bred from a male sterile mutant discovered by Osman and Yermanos [21], with the male sterility controlled by a recessive gene, *Sims2* [8]. 95ms-5 was bred by ^60^Co gamma ray treatment of seeds in cultivar Yuzhi No.4 [22], and its fertility was controlled by a single recessive gene, *Sims1* [8]. 0176A was derived from a mutant in a landrace from Anhui Province, China; one or two major recessive genes and several minor genes controlled its male sterility [20]. More recently, a stable, monogenic RGMS line (D248A) was developed from male sterile mutants discovered in the variety Zhuzhi 4, which had great potential for the breeding of hybrid varieties [11]. However, all the above RGMS lines cannot directly produce a population with 100% sterile plants for hybrid seeds production. Instead, the RGMS systems usually produce a population with 50% male sterile plants, which can be used for hybrid seeds production, and 50% fertile plants, which should be removed before flowering. This process is labor-intensive, time-consuming, and cost-inefficient. Nevertheless, in comparisons with other currently available approaches, RGMS is still the most valuable way for sesame hybrid seed production. Furthermore, the shortcoming of RGMS can be compensated by the value added by strong heterosis. So far, several hybrid varieties (i.e. Yuzhi 9, Zhongzazhi 1, Zhongzazhi 2, Wanzhi 1) have been released and grown in China.

Although importance, very few cytogenetic and molecular studies have been carried out on sesame RGMS, thus hindering the effective manipulation and utilization of such system. A pilot cytogenetic study on ms86-1 showed that microspore abortion occurred at early microspore stage, and the abnormal tapetum was the direct cause of pollen abortion [23]. However, this study provided little information for the underlying mechanism. Later, ms86-1 was further investigated by transmission electron microscope (TEM), which indicated that male sterility might arise at the microsporocyte formation stage, and a number of facts might lead to the male sterility: irregular shapes of the microsporocyte’s cell walls, distortion of cell walls at meiosis stage, failure to form probaculums on the outside of the plasma membrane of microsporocytes, abnormal villiform deposits on the outside of the callose wall during tetrads stage, disintegration of tetrads cells, and less secretion of ubisch bodies [24]. Anther development on 95ms-5 was also investigated using paraffin method. It was shown that collapsed microspores, expanded vacuoles as well as delayed degeneration of tapetum at the pollen maturation stage were all closely associated with the abnormal male gametogenesis at the mononuclear microspore stage [25]. Moreover, Cytological investigation demonstrated that pollen abortion in W1098A plants (a dominant GMS line) began in pollen mother cells, continued throughout pollen development, and peaked at the late microspore stage. The abortion process was closely associated with the abnormal behavior of the tapetum [12].

With the development of molecular marker technology, a few studies were reported on the molecular mapping of agronomically important traits in sesame, which would facilitate the transferring and pyramiding of male sterile genes with other elite traits in different backgrounds for new RGMS lines, or the identification and removal of 50% male fertile plants within the RGMS lines before flowering if there is no visible linked marker trait. Recently, markers linked to important agronomic traits such as yield, oil content and seed coat colour were identified either by QTL mapping or by genome wide association mapping in sesame [26,27]. Moreover, the gene conferring RGMS was also mapped within an 8.0 cM interval defined by two AFLP markers, P06MG04 and P12EA14 [8]. Two SSR markers (SBM298 and GB50) were identified on both sides of a dominant male sterile gene in a DGMS line (W1098A) at a respective genetic distance of 0.15 and 0.70 cM [12]. However, both cytological and molecular studies on a newly developed sesame RGMS line (D248A) are still lacking.

The objectives of this work were to elucidate the cytological and genetic basis of the male sterility in the newly developed RGMS line, D248A. We investigated the cause of pollen abortion in this line using light and electron microscope. To obtain a 100% male sterile population for hybrid production, we identified several molecular markers linked to the genic male-sterile gene. The results of this study will facilitate the breeding of hybrid variety and deepen our knowledge on the mechanism of male sterility in sesame.

## Materials and methods

### Plant materials

The sesame plant materials used in this study were the homozygous RGMS line D248A and its heterozygous fertile maintainer line D248B, which were bred from a spontaneous male sterile mutant discovered in cultivar Zhuzhi 4 through consecutive sib-mating between segregated sterile and fertile plants. A preliminary genetic study has shown that it was controlled by a single recessive gene *ms* [11]. Sterile D248A (genotype *msms*) was maintained by sib-mating with fertile D248B (*Msms*) for more than 9 generations. Except for pollen fertility, D248A and D248B were almost identical thus can be regarded as near isogenic lines (NILs). All plants were grown in the experimental farm at the Oil Crops Research Institute, the Chinese Academy of Agricultural Sciences, Wuhan, China (113′53″ E, 29′58″ N). Field management followed the normal practical procedures during the growth season (from May to October). The fertility of the plants was initially determined by naked eye at early flowering stage in the field, and then further examined in the laboratory at full blossom stage.

### Pollen fertility investigation

To characterize the pollen fertility of D248A reliably, we used three complementary methods, namely acetate magenta staining, FDA (fluorescein diacetate) staining, and semisolid suspension medium cultivation. At full-blossom stage, pollen grains were collected from dehiscent flowers of ten D248A plants and ten D248B plants in the morning (6:00–7:00), spread onto slides and stained by 1.0 g/L acetocarmine for 2 min or by FDA for 5 min at room temperature, and then carefully examined under light microscope (OLYMPUS, BX-61) or fluorescence microscope (Leica DMi8 Fluorescence Imaging System), respectively, as described before [28]. A microprojector was used to determine pollen stainability percentage at ×200 magnification. Fields of 30–40 well separated grains were classified, with three replicates. Individual plants were recorded as either fertile or male sterile, based on pollen stainability. Fully developed, red-stained (detected by acetocarmine) or strong fluorescent-emitting (detected by FDA) pollen grains were classified as fertile, while shriveled, unstained or lightly stained, weak fluorescent-emitting pollen grains were scored as sterile.

Pollen germination on semisolid suspension medium was carried out as previously described [11,29]. In brief, semisolid suspension medium was prepared by mixing 10% (w/v) sucrose (C_12_H_22_O_11_), 0.4% (w/v) purified agar, 0.1% (w/v) calcium nitrate [Ca(NO_3_)_2_·4H_2_O], 0.01% (w/v) boric acid (H_3_BO_3_) and adjusted to pH 7.0 with 0.1 mol/L potassium hydroxide (KOH) solution. The medium was autoclaved, poured into 9-cm petri dishes, cooled down to room temperature and covered to prevent evaporation for overnight before use. Pollen grains from each genotype were inoculated on this medium for 60 min at room temperature before examining pollen viability under microscope. Three counts of 100 grains were carried out after inoculation. Average pollen germination rate was calculated from all counts for a line.

#### Paraffin sectioning

Based on previous research, the development process of sesame anther could be tentatively divided into 5 stages, i.e. sporogonium stage (with the bud size <2 mm), microspore mother cell (MMC) stage (bud size 2–3 mm), tetrads stage (bud size 3–5 mm), microspore stage (bud size 5–25 mm) and mature pollen stage (bud size >25 mm), respectively [11]. When flowering, buds at five developmental stages, according to their bud sizes, were collected from D248A and D248B plants and immediately fixed in FAA solution (90 ml 70% alcohol, 5 ml acetic acid, 5 ml formalin) for over 24 h. Buds were vacuumed to eliminate any air inside samples, stained with Love’s hematoxylin or safranin O-fast green, sliced into 5-μm-thick sections and then mounted onto slides with neutral gum. Stained sections were observed and photographed with light microscope (OLYMUS BX-61, Japan).

#### Scanning electron microscope (SEM) observation

For SEM observation, anthers were collected at mature pollen stage and immersed in 3% glutaraldehyde (in 0.1 Mol/L phosphate buffer, pH 7.2) under 4°C for 7 d. The anther samples were then dehydrated with increasing concentration of ethanol series (30%, 50%, 70%, 80%, 90% each for 30 min; 100% twice for a total of 60 min), followed by a final 30-min washing with isoamyl acetate. Before observation in a SEM (S-450, Hitachi, Japan), anther samples were further dried out by critical-point drier (CPD-030, Balzers) and covered with gold using a sputter coater (EikoIB5, Hitachi, Japan) [30].

#### Molecular marker analysis

To generate a segregating population for the genetic mapping of fertility, a male-sterile plant from the near-isogenic line D248A was crossed with its fertile counterpart (D248B). The resulting sib-mating NIL population, consisting of 372 plants, was expected to only exhibit segregation at the genetic loci controlling pollen fertility.

Genomic DNA was extracted from young leaves [31]. DNA concentration was adjusted to 50 ng/μl. Equal amount of DNA from 10 fertile and 10 sterile plants randomly selected from the NIL population were pooled to construct the fertile (BF) and sterile (BS) bulks, respectively. Primer sources were from the literatures [32–34]. A total of 79 InDel markers and 1500 SSR markers were used in the present study. PCR amplification was carried out in a volume of 10 μl including 10 ng each of forward and reverse primers and 50 ng of genomic DNA. PCR cycling parameters were as follows: 94°C for 30 s, 58°C for 40 s, and 72°C for 50 s, 37 cycles with the annealing temperature touched down by 0.7°C per cycle for the first ten cycles. PCR products were separated on 6% denaturing polyacrylamide gel and visualized using the silver staining method [12].

PCR fragments showing polymorphisms between BF and BS was repeated, and their Linkage with male sterile trait were confirmed by analyzing 20 individual plants constituting BF and FS. These confirmed markers were further analyzed in the whole NIL population (n = 372). Polymorphic bands were scored either as dominant or co-dominant locus. A local linkage map was constructed with MapMaker v3.0 [35]. A LOD score of 3.0 was used as threshold for map construction. Recombination frequency was transformed into genetic distance (centimorgans, cM) using Kosambi’s mapping function [36].

To fine map fertility trait, the identified markers were anchored to the updated sesame pseudo-chromosomes by BLAST searching, and new set of markers surrounding *Ms* locus were designed using the online source SisatBase [37] and subject to population screening and linkage analysis. Primer sequences for linked markers were shown in Table 1.

**Table 1:** Pollen fertility investigated by different methods.

Genotype	Acetocarmine ^a^	FDA ^b^	Semisolid medium ^c^
D248A	4.6 ± 1.4	2.9 ± 0.8	0
D248B	99.4 ± 0.8	98.6 ± 0.6	92.3 ± 2.6

^a^ Mean and standard deviation of percentage of round, filled and fully acetocarmine-stained pollens.

^b^ Mean and standard deviation of percentage of round pollens with strong fluorescent emission.

^c^ Mean and standard deviation of percentage of pollens germinated in medium.

## Results

### Pollen fertility of D248A

**Fig 1:**
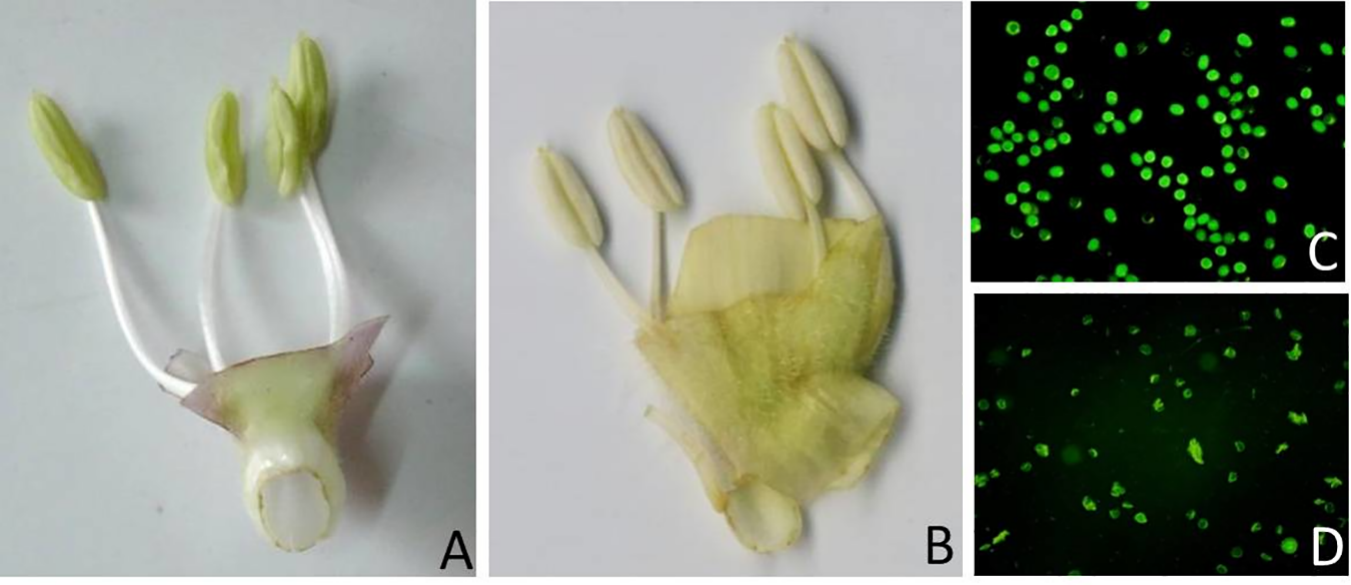
**Anther morphology and pollen fertility of D248A (A, D) and D248B (B, C).** (a, b) Anther, (c, d) Pollen grain stained by FDA. Magnitude power was set to ×100 for (c-f).

The first flowering and seed setting day was similar for D248A and its wild type (i.e. Zhuzhi 4) in Wuhan growing condition. However, the flowering period was 10–12 days longer for D248A which enable a 79–95% increase of seed yield per plant [11]. When flowering, D248A plant exhibited green and slim anther (Fig 1A), while D248B plant showed white and plump one (Fig 1B). Other than male sterility, there were no different phenotypes. Pollen fertility was examined by acetocarmine staining, FDA staining and semi-solid suspension medium cultivation. For acetocarmine staining, only 4.6% of the D248A pollens were round, filled, and stained, while the value was determined as 99.4% for D248B (Table 1). FDA staining showed a similar result; 98.6% of D248B pollen grains were plump and round, which could also emit strong inflorescent green light, a sign of good viability and fertility (Table 1, Fig 1C). However, the pollen fertility of D248A was only 2.9%; most of the D248A pollens were irregular in shape or even empty and could only emit faint inflorescent light (therefore were recorded as ‘male sterile’) (Fig 1D). For semi-solid suspension cultivation, 92.3% of D248B pollen grains could germinate after 60 min of incubation at room temperature (Table 1); of these, around 10% could produce long pollen tube (3–5 times of the pollen grain diameter). But in D248A anther, no pollen grains could germinate; the round-shape pollens, if any, were crinkled and in plasmolysis, with much less contents inside. Taken together, the above results indicated that D248A was highly male sterile. Although a few round pollen grains were occasionally observed [11], they could not germinate in the semi-solid suspension medium, and therefore were regarded as abortive.

**Fig 2:**
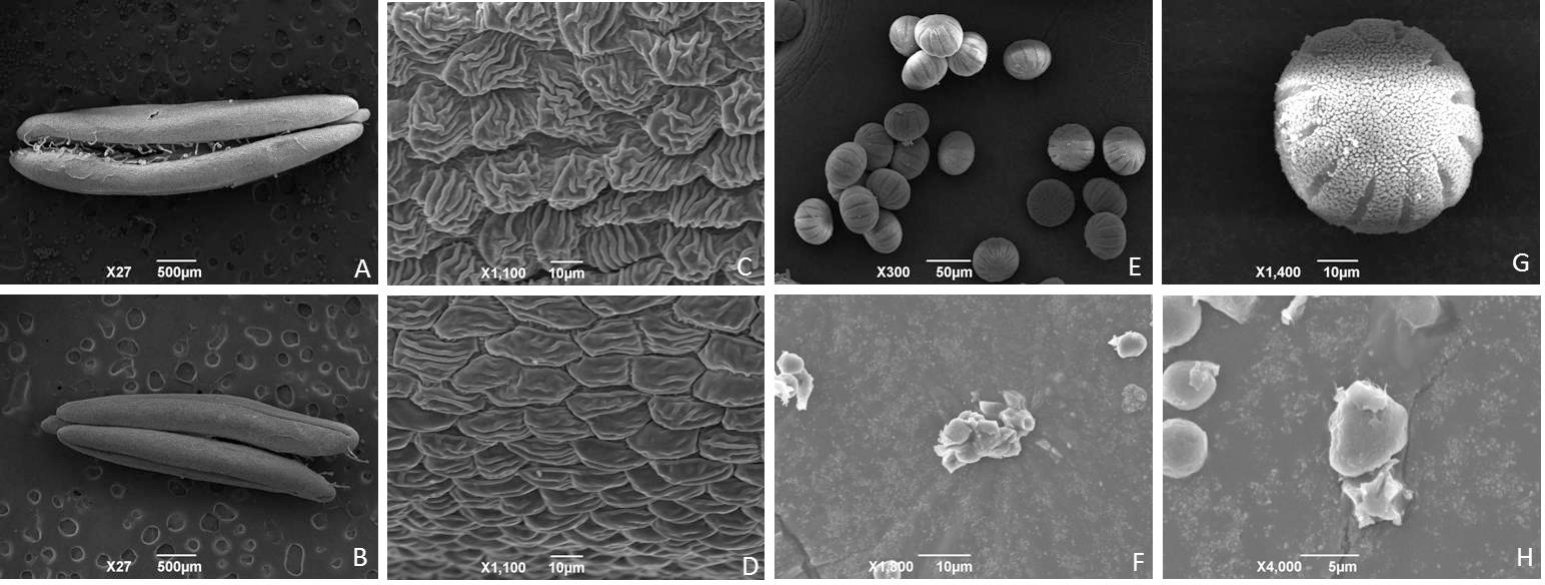
Cytological characterization of anthers and pollen grains using scanning electron microscopy. (a, c, e, g) D248B, (b, d, f, h) D248A. (a, b) anther, (c, d) xx, (e, g, f, h) pollen.

Pollen morphology was further characterized using SEM. The pollen sac in D248B could split, which disclosed plenty of normal pollens inside (Fig 2A and 2E). However, the D248A pollen sac could not split (Fig 2B), and very few pollen grains could be observed inside, if squeezed (Fig 2F). Relative to the small and slightly shrunk three-dimensional reticular structure formed on D248A epidermal cells (Fig 2D), those in D248B were big and markedly shrunk (Fig 2C). Generally, D248B pollen grains were bigger and plump, with 12–15 overt furrows on surface (Fig 2G). In contrast, those of D248A were smaller, irregular in shape, and even adhered to one another (Fig 2H). Thus, the highly abortive morphology of D248A pollen grain was confirmed at ultrastructure level.

### Comparison of anther development

To investigate the cytological mechanism underlying anther abortion, we used light microscope to observe and compare the anther development in D248A and D248B. The developmental process of sesame anther was tentatively divided into five stages, i.e. the microsporocyte formation, MMC, tetrads, microspore, and pollen maturation stages.

#### Microsporocyte formation stage

**Fig 3:**
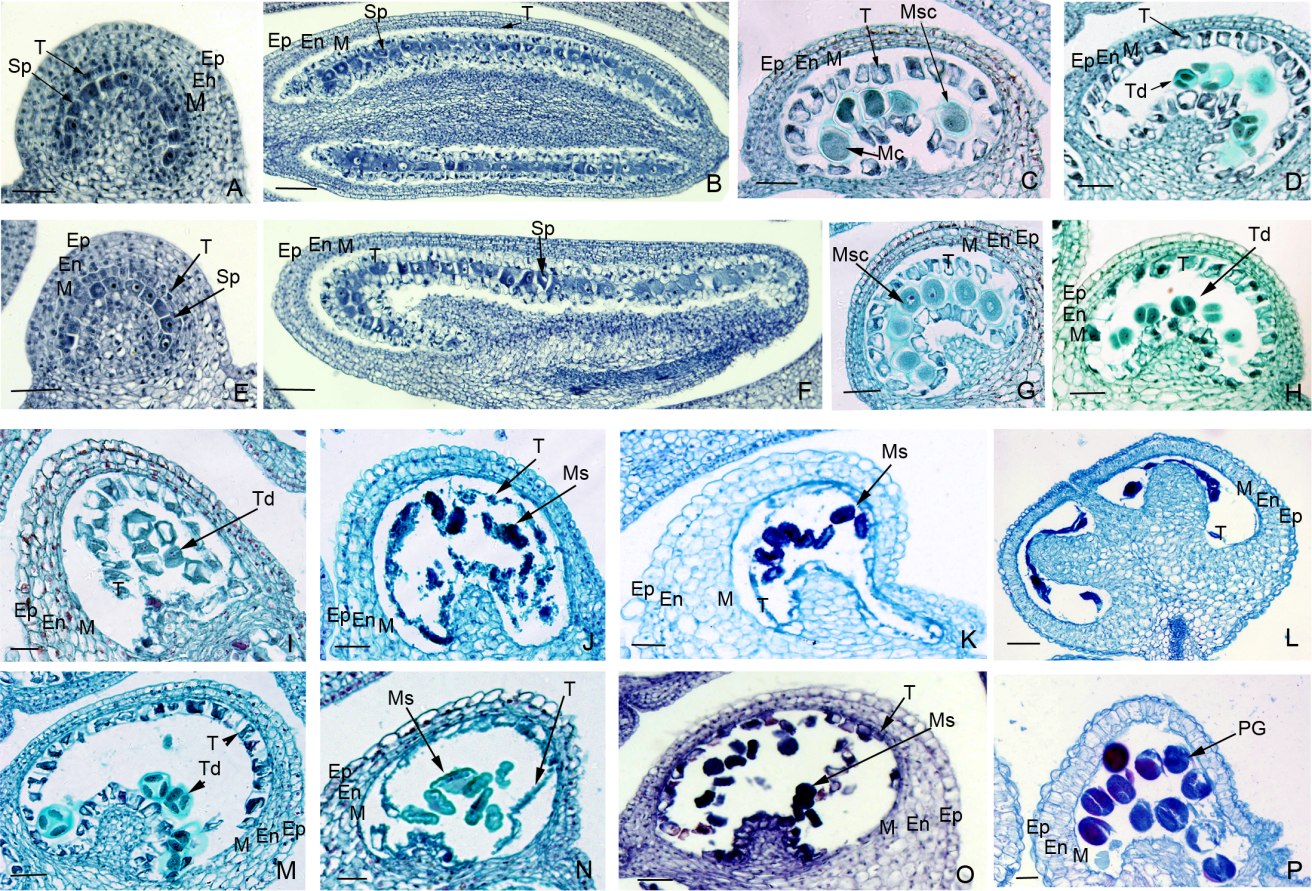
**Micrographs of anther development in D248A (A-D, I-L) and D248B (E-H, M-P) using light microscopy.** (a, b, e, f) sporogonial stage. (c, g) microspore mother cell stage. (d, h, i, m) tetrads stage. (j, k, n, o) microspore stage. (l, p) mature pollen stage. Abnormality of anther development began at the tetrads stage (d, i) and peaked at the microspore stage (j, k) as evidenced by the deformation and degeneration of both the tapetum and microscope. En endothecium, Ep epidermis; M middle cell layer; Sp sporogenous cell, Ms microspore, Mc meiotic cell, Msc microsporocyte; PG pollen grain, T tapetum, Td tetrads. Bars = 60 μm. The magnification power was 10×20 in b and f, 10×40 in a, c-e, h-p.

At the sporogonial stage, the normal stamen primordium locating in four corners began to divide asymmetrically, then formed the butterfly-like chambers, if observed on transverse section. Each chamber comprised anther wall and one layer of sporogenous cells. The anther wall consisted of four layers, which were, from outer to inner, epidermis, endothecium, middle cell layer, and tapetum, respectively (Fig 3A). All cells of anther wall were similar in size and closely connected to each other. However, the sporogenous cells were notably bigger than anther wall cells. They always exhibited a deeper color after staining, with overt dense cytoplasm and nucleus. The numbers of sporogenous cells varied in chambers (from 4 to 13) and they were arranged into a curve on transverse section (Fig 3A and 3E) or a line on longitudinal section (Fig 3B and 3F). No obvious difference was observed for sporogenous cells between D248A and D248B.

#### MMC stage

At this stage, the microsporocytes of D248B were large, detached, and orderly arranged, with one or two nuclei within each cell (Fig 3G). The tapetal cells tightly surrounding the microspore mother cells would gradually separate from the middle cell layer. However, compared with D248B, the developing anthers of D248A appeared abnormal soon after the formation of microsporocytes. Although some of the D248A microsporocytes were like those of D248B (large, separated yet in neat arrangement), most of the others had less cytoplasm and no obvious nucleus, and were irregular in shape or even shrunk (Fig 3C). Notably, the tapetal cell development was seemingly normal up to this stage, given the fact that there was no obvious difference from D248B.

#### Tetrads stage

With the development of microspore mother cell, tetrads spores were formed shortly after meiotic division in D248B (Fig 3H). These spores were surrounded by callose and rich in cytoplasm and organelles. Meanwhile, tapetal cells began to degenerate normally; the cytoplasm within tapetal cell was further reduced and condensed (Fig 3M). In contrast, meiosis could not carry out normally in D248A microspore mother cells, leading to the formation of much less normal cell-size tetrads spores (Fig 3D). Moreover, abnormalities such as condensation and vacuolation in cytoplasm were observed in D248A tetrads (Fig 3I). Besides, tapetal cells degeneration was also delated in D248A.

#### Microspore stage

D248B tetrads could easily free from callose and separate into individual cells, which gradually grew up into microspores. The microspores, which were just released from callose, always exhibited condensed and darkly dyed cytoplasm and were irregular in cell shape (Fig 3N). After further development, the microspores, although their cytoplasm decreased, became bigger and bigger and were round in shape (Fig 3O). Meanwhile, tapetal-cell layer was degraded rapidly during the formation of microspore and was almost completely disappeared at the late-mononuclear stage, leaving only some debris (Fig 3O). In comparison, the tetrads in D248A were irregular in shape and deeply dyed; most of them could not release from callose (Fig 3J) and develop normally. The resulting microspores became crumpled, condensed, empty, and gradually losing their shape (Fig 3J and 3K). Contrast to the nearly complete disintegration in D248B (Fig 3O), tapetal cells in D248A were disintegrated at a much slower rate and had abundant unexhausted cytoplasm (Fig 3J and 3K).

#### Mature pollen stage

Following two successive divisions of a microspore, mature pollen grains were formed in D248B and began to be released. The tapetal cells were completely degraded and their debris were hardly observed (Fig 3P). However, almost all D248A pollen grains, if any, were empty and abortive, yet leaving some viewable tapetal cells debris (Fig 3L). At this stage, many D248A anther wall cells were also distorted and lost their normal shape.

### Mapping of male sterility gene

A NIL population comprising 372 plants (212 fertile and 160 sterile) was used for genetic mapping of the *Ms* locus in D248A. Seventy-Nine InDel markers and 1500 SSR markers were screened against the parents and the two gene pools simultaneously, which typically yielded 1–5 bands, depending on the primer used. One InDel (SB2993) and one SSR (LG1-179) on chromosome 1 was polymorphic both in the parent and the DNA pools. To verify their linkages with *Ms* gene, SB2993 and LG1-179 were further checked in 10 fertile and 10 sterile individuals constituting the two bulks. Most of the bands appeared in fertile plants but not in sterile plants, which is consistent with the genome constitution of fertile (*Msms*) and sterile plants (*msms*) in the NIL population. There were none and only two recombinants for SB2993 and LG1-179, respectively, thus indicating the reliability of the marker-trait association. To fine map this gene, we downloaded the sesame genomic sequence within 1000 kb upstream and downstream of the closest marker SB2993 and designed a new set of 179 primer pairs. These primers were again tested in the same parents, bulks, and 20 individuals. Reproducible polymorphisms were observed for marker LG1-111, LG1-170 and LG1-176.

**Fig 4:**
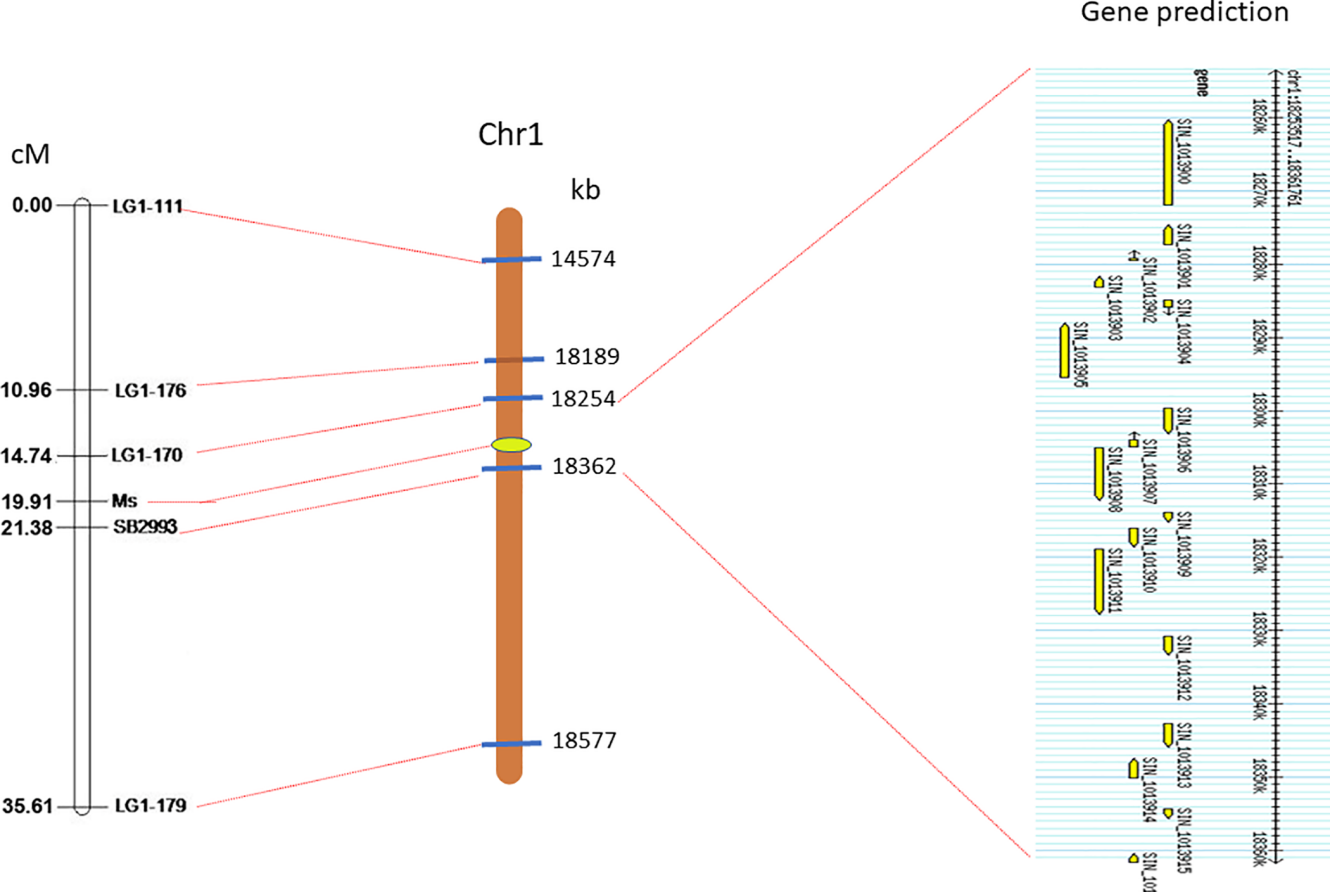
Genetic and physical mapping of the male sterility locus (*Ms*) in D248A. Sesame genome sequence version 2.0 (http://www.sesame-bioinfo.org/Sinbase2.0/) was used for physical mapping and gene prediction.

To construct a map for *Ms* gene, we genotyped the entire NIL population using the 5 polymorphic markers (Table 2). A local genetic linkage map was constructed, and the *Ms* gene was found to reside between SB2993 and LG1-170, with respective genetic distance of 1.47 and 5.17 cM. Another closely linked marker, LG1-176, was in the upper part of the linkage group and 8.95 cM away from the *Ms* locus. The other two markers (LG1-111 and LG1-179) were loosely linked with the trait and located at the opposite ends of the linkage group (Fig 4).

**Table 2:** Primers sequences for markers linked to male sterility in D248A.

Primer code	Forward sequence (5’-3’)	Reverse sequence (5’-3’)
LG1-179	TGGAGGGCTACTGAAGATTA	CGTAAGAGAAATCCAAGCAT
LG1-111	AACTGGACTGACACCAAATG	CTGTCTCCACTTTCTTTTGT
SB2993	GTTTCCGACCACGCTTCCTA	GCTCGTCAATGGAGCTAGGG
LG1-170	TGGTGCTTTCTTTGGTAGAT	GATTTGTTGACCCCAACTTA
LG1-176	AATTCCCCTGTTTATTACCC	CCTCGTTTTATCTGTTTGCT

With the availability of sesame whole genome sequence, we further anchored all the five markers onto the downstream of chromosome 1, at physical position 14574–18577 kb, based on the updated sesame genome sequence v2.0 (http://www.sesame-bioinfo.org/cgi-bin/gb2/gbrowse/Sinbase2.0/). The *Ms* gene was located within a 108-kb interval (physical position at 18254–18362 kb) defined by marker LG1-170 and SB2993. Within this genomic region, 15 gene models were predicted and only 10 had GO or InterPro annotations (Table 3). These genes were predicted to involve in a wide range of activities such as transferase, oxygenase, or transcription factors.

**Table 3:** Candidate genes for the male sterility in D248A.

Gene ID ^a^	Gene Ontology Annotation	InterPro Annotation
SIN_1013900	Metal ion binding	Zinc finger, RING-type
SIN_1013901	null	Alpha/Beta hydrolase fold
SIN_1013903	transferase activity	Thiolase-like
SIN_1013905	ATP binding	Helicase superfamily, ATP-binding domain
SIN_1013906	Oxidoreductase activity	Carotenoid oxygenase
SIN_1013909	Transferase activity, transferring hexosyl groups	UDP-glucuronosyl/UDP-glucosyltransferase
SIN_1013911	GTPase activity	Small GTP-binding protein domain
SIN_1013913	DNA binding	Transcription factor, SBP-box
SIN_1013914	DNA binding	Transcription factor, SBP-box
SIN_1013915	Peptidase M10, metallopeptidase	Metalloendopeptidase activity

^a^ gene prediction and annotation were carried out in the online database (http://www.sesame-bioinfo.org/cgi-bin/Sinbase2.0/genedetail.cgi?locus=SIN_1013902)

## Discussion

D248A was a new sesame RGMS line developed from a spontaneous male sterile mutant discovered in Zhuzhi 4, an old cultivar released in 1978 (a time when no GMS was available in China). It exhibited very good agronomic traits such as vigorous growth, long blossom duration, complete male sterility on the sterile plants, and higher seed yield, which made it a valuable stock for hybrid variety breeding [11]. Besides, there also existed some other RGMS lines. For instance, ms86-1 was bred by Tu et al. [18] from the original male sterile mutant introduced from Osman and Yermanos [21], and 95ms-5 was derived from gamma ray treatment of cultivar Yuzhi 4 [19]. Based on the independent genetic origin of these RGMS lines, we argued that D248A was novel, although direct evidences such as allelic test is still needed to determine their genetic relationships.

Cytogenetic study had disclosed some key features contributing to male sterility in D248A (Fig 3). Sporogenous cells were normal at microsporocyte formation stage but began to abort at MMC stage and persisted until microspore stage. The microsporocytes had less cytoplasm and no obvious nucleus. Normal meiosis failed in microspore mother cells, from which smaller and irregular tetrads were produced. Later, the tetrads further experienced cytoplasm condensation and vacuolation and could not release from callose. The resulting microspores became crumpled and condensed, and gradually lost their cell shapes. Moreover, tapetal cells degeneration was also delated and incomplete, leaving abundant unexhausted cytoplasm. In addition, many anther wall cells were also distorted and lost their normal shape. All the above abnormalities were likely to be responsible for pollen abortion in D248A. Our observations were in accordance with previous cytogenetic studies on sesame GMS lines, where delayed and incomplete disintegration of tapetum cells were important factors contributing to anther abortion [12, 23–25]. However, there were still some notable differences. In contrast to the abortion beginning at MMC stage in D248A, microsporogenesis study of ms86-1 indicated that male sterility might arise much earlier, in microsporocyte formation stage. The cell wall of the sterile microsporocytes was also irregular during their formation stage [24]. For 95ms-5, abnormal inhibited male gametogenesis appeared during the mononuclear microspore stage [25], which was later than D248A. Taken together, it seems that D248A employs a different cytogenetic mechanism.

Development of molecular markers closely linked to male sterility could greatly facilitate the transferring of *ms* allele into other elite lines or developing a 100% sterile population for hybrid seed production. We have identified 5 markers for *Ms* gene in D248A using a NIL population (Fig 4). The two closest markers (SB2993 and LG1-170) at opposite sites, were only 1.47 and 5.17 cM away from the target gene and could be used directly in marker-assisted breeding of new hybrid variety via backward selection. Furthermore, by BLAST search using the linked marker sequences, we could further anchor the gene responsible for male fertility into a 108-kb fragment in the downstream of chromosome 1, where only 15 candidate genes were predicted (Fig 4). These findings provided an opportunity not only to develop co-segregating functional markers, but also isolate the male sterility gene. Previously, Zhao et al have identified 9 AFLP markers linked to the male sterile gene *SiMs1* in sesame RGMS line 95ms-5, which included a co-segregated marker, P01MC08 [8]. However, they failed to locate the *SiMs1* gene into a specific chromosome, due to the lack of whole genome sequence at that time. More recently, 13 SSR markers were found to be linked to the *Ms* locus in the sesame dominant GMS line W1098A. The closest markers on both sides of *Ms* were SBM298 and GB50, at a respective genetic distance of 0.15 and 0.70 cM [12]. After the release of sesame whole genome sequence [38], the *Ms* gene in W1098A was shown to reside in chromosome 7, according to the physical position of marker SBM298 (chr7:14405910…14405773) (http://www.sesame-bioinfo.org/Sinbase2.0/markers.htm). Thus, the different location of *Ms* gene in D248A further strengthened its novelty.

The present study also provided a shortlist of 15 candidate genes for *Ms* in D248A. Among these, 10 have annotations and the other five were functionally unknown (Table 3). The main activities of these ten genes involved in zinc finger, hydrolase folding, thiolase-like transferase, ATP-binding domain, carotenoid oxygenase, GTPase, transcription factor, SBP-box, and metallopeptidase. As demonstrated by cytogenetic study, anther development is a complex process, and any failure of this process will lead to male sterility [39]. It is possible that any breakdown of the shortlisted genes will affect the pollen fertility. Among these, four genes were highlighted. SIN_1013913 and SIN_1013914 were annotated as a SBP-box transcription factor. In Arabidopsis, SBP-box genes were also known as SPL genes which encode a plant-specific family of transcription factors binding DNA [40]. A recent study showed that the SBP-box transcription factor SPL8 was required for promoting sporogenous cell and parietal cell formation in early anthers; loss-of-function of *SPL8* would result in semi-sterile phenotype [41]. Another gene of interest, SIN_1013909 encodes UDP-glucuronosyl/UDP-glucosyltransferase. The Arabidopsis homolog gene (UGT79B6) was localized in tapetum cells and microspores, which encoded a key modification enzyme for determining pollen-specific flavonol structure that affected pollen fertility [42]. Despite the lack of evidence for the influence of UGT79B6 on pollen fertility in Arabidopsis, studies in petunia and maize had shown that mutants deficient in flavonoids were unable to germinate pollen tubes, resulting in male sterility [43,44]. The fourth sesame gene of interest (SIN_1013911) encodes a GTP-binding protein and involves in GTPase activity. In wheat, yeast two-hybrids (Y2H) analysis showed that GTP-binding protein (GTPase) in the S2-stage spikes significantly interacted with the *Ms2* gene conferring male sterility [45]. Therefore, this gene would have a similar role in sesame anther development. In summary, this work produced some very interesting markers or new genes and provided a deeper insight into male gametogenesis in sesame.
